# Web-Based Measure of Life Events Using Computerized Life Events and Assessment Record (CLEAR): Preliminary Cross-Sectional Study of Reliability, Validity, and Association With Depression

**DOI:** 10.2196/10675

**Published:** 2019-01-08

**Authors:** Antonia Bifulco, Ruth Spence, Stephen Nunn, Lisa Kagan, Deborah Bailey-Rodriguez, Georgina M Hosang, Matthew Taylor, Helen L Fisher

**Affiliations:** 1 Centre for Abuse and Trauma Studies Department of Psychology Middlesex University London United Kingdom; 2 Wolfson Institute of Preventive Medicine, Centre for Psychiatry Barts & The London School of Medicine & Dentistry Queen Mary University of London London United Kingdom; 3 Department of Psychosis Studies Institute of Psychiatry, Psychology & Neuroscience King's College London London United Kingdom; 4 Social Genetic & Developmental Psychiatry Centre Institute of Psychiatry, Psychology & Neuroscience King's College London London United Kingdom

**Keywords:** depression, life change events, life stress, health technology, internet, psychometrics, psychological tests

## Abstract

**Background:**

Given the criticisms of life event checklists and the costs associated with interviews, life event research requires a sophisticated but easy-to-use measure for research and clinical practice. Therefore, the Computerized Life Events and Assessment Record (CLEAR), based on the Life Events and Difficulties Schedule (LEDS), was developed.

**Objective:**

The objective of our study was to test CLEAR’s reliability, validity, and association with depression.

**Methods:**

CLEAR, the General Health Questionnaire, and the List of Threatening Experiences Questionnaire (LTE-Q) were completed by 328 participants (126 students; 202 matched midlife sample: 127 unaffected controls, 75 recurrent depression cases). Test-retest reliability over 3-4 weeks was examined and validity determined by comparing CLEAR with LEDS and LTE-Q. Both CLEAR and LTE-Q were examined in relation to depression.

**Results:**

CLEAR demonstrated good test-retest reliability for the overall number of life events (0.89) and severe life events (.60). Long-term problems showed similar findings. In terms of validity, CLEAR severe life events had moderate sensitivity (59.1%) and specificity (65.4%) when compared with LEDS. CLEAR demonstrated moderate sensitivity (43.1%) and specificity (78.6%) when compared with LTE-Q. CLEAR severe life events and long-term problems were significantly associated with depression (odds ratio, OR 3.50, 95% CI 2.10 to 5.85, *P*<.001; OR 3.38, 95% CI 2.02 to 5.67, *P*<.001, respectively), whereas LTE-Q events were not (OR 1.06, 95% CI 0.43 to 2.60, *P*=.90).

**Conclusions:**

CLEAR has acceptable reliability and validity and predicts depression. It, therefore, has great potential for effective use in research and clinical practice identifying stress-related factors for the onset and maintenance of depression and related disorders.

## Introduction

### Background

Severe life events and chronic long-term problems are significant factors in the onset and maintenance of depression and various clinical disorders [1-6] and an important focus of etiological research. However, life events research has become overreliant on quick-to-administer self-report checklists, resulting in a loss of specificity and power [7].

### Life Event Checklists

Checklist approaches suffer from methodological limitations. The life events involved comprise predetermined event-types to endorse, without personal context, making them reliant on subjective interpretation, with potential for stress-response and stress-outcome confusion [7]. Event dating, severity, and independence are absent, despite being critical for determining their role as “provoking agents” prior to the onset of psychological disorders. Checklist life events lack information on focus and fall prey to “intracategory unreliability,” having no definition or benchmark for guidance [8]. Additionally, checklist approaches condense linked events (eg, solely endorsing a birth event, which can include antenatal and postnatal experience).

### Life Event Interviews

Life event interviews overcome these methodological constraints and are viewed as more reliable and accurate [9]. The Life Events and Difficulties Schedule (LEDS) [10], often considered the gold standard [11], is a widely used semistructured interview. It captures context and utilizes investigator-based ratings of severity according to precedents to reduce subjective bias [7]. The LEDS is better than self-report measures at capturing life events [12], and its severe life events (those with high negativity after 10-14 days and focused on the self) show superior effect sizes for depression [13]. Those prior to depression onset are given particular attention as provoking agents [14,15].

However, interviews are a high cost in both time and the need for expert administration and lack feasibility for large dispersed samples [16]. Furthermore, where research relies on checklists, in large-scale projects investigating gene-environment interactions, findings are mixed, for example, Culverhouse et al and Risch et al [17,18], with checklists identified as a key factor in nonreplication [19,20].

### A New Life Events Measure

The need for a new reliable and valid life stress measure of life events that is less time- and cost-intensive than an interview but improves on checklists, is approached here through technological Web-based advances, which are increasingly popular in researching psychological concepts and disorders [21-23]. The Computerized Life Events and Assessment Record (CLEAR) is a Web-based measure of life events, including severe life events and long-term problems. The design was influenced by the LEDS to capitalize on the many benefits of the interview and improve existing self-report measures [16]. Advantages include using precoded algorithmic scoring [24], lower costs [25], project-personalized presentation [26], and personalized feedback [27]. To date, such digitalized methods have not been applied to the assessment of complex social risk factors such as life events.

This paper aims to assess the psychometric properties of CLEAR. Test-retest reliability was assessed over 4 weeks; concurrent validity was checked against parallel LEDS interviews and a self-report checklist, and predictive validity involved investigating associations between CLEAR severe life events and depression.

## Methods

### Participants

The sample consisted of 126 students (mean age 20.5 (SD 0.35) years; range 18-46 years) recruited from Middlesex University and 202 midlife adults (mean age 57.6 (SD 7.87) years; range 36-75 years) recruited from the Depression Case Control (DeCC) sample (75 recurrent depression cases and 127 controls). There were more females overall. Due to the prior genetic sampling, the DeCC participants were all white, while the students were more likely to be from ethnic minorities. Most of the DeCC sample had partners and children and were educated to at least a degree level. Few students had children, and over half had partners. The DeCC clinical group had the highest rate of current depression, which significantly differed from the control sample. The student rates proved to be more similar to the clinical group (Table 1).

#### Reliability Subsample (n=61)

Test-retest reliability of CLEAR was undertaken on a subset of the main sample (20 DeCC depression cases, 21 DeCC control group, and 20 students) measured 3-4 weeks apart.

#### Validity Subsample (n=30)

A subsample of 30 participants (10 DeCC depression cases, 10 DeCC controls, and 10 students) completed CLEAR and the LEDS interview, with half completing either CLEAR or LEDS first.

### Procedure

The DeCC sample was drawn from a UK multicenter case-control genetic association study of unipolar depression in midlife white respondents [28,29]. Depressed patients were originally identified through psychiatric clinics, hospitals, general medical practitioner surgeries, and media advertisements, and had experienced at least 2 episodes of unipolar depression. Matched controls were recruited through general medical practices across the United Kingdom and were excluded if they had a personal or first-degree relative with a history of psychiatric disorder (Korszun et al) [29].

**Table 1:** Demographic characteristics by group (N=328).

Characteristics	Total DeCC^a^ (n=202), n (%)	Controls (n=127), n (%)	Clinical (n=75), n (%)	DeCC comparison χ^2^_1_	*P* value	Students (n=126), n (%)	DeCC comparison χ^2^_2_	*P* value
Gender: female	122 (60.4)	70 (55.1)	52 (69.3)	4.0	.05	112 (88.9)	30.8	.001
Ethnicity: white	202 (100.0)	127 (100.0)	75 (100.0)	N/A^b^	N/A	47 (37.3)	162.8	.001
Degree-level education	103 (51.0)	66 (52.0)	37 (49.3)	0.1	.79	13 (10.3)	60.5	.001
In work	125 (61.9)	91 (71.7)	34 (45.3)	13.9	.001	61 (48.4)	5.7	.02
Partnered	167 (82.7)	113 (89.0)	54 (72.0)	9.5	.002	67 (53.2)	33.0	.001
Partner in work	104 (51.5)	68 (53.5)	36 (48.0)	1.2	.27	41 (32.5)	0.2	.70
Has children	164 (81.2)	110 (86.6)	54 (72.0)	6.6	.01	5 (4.0)	185.3	.001
General Health Questionnaire depression	43 (21.3)	10 (7.9)	33 (44.0)	37.5	.001	44 (34.9)	6.8	.01

^a^DeCC: Depression Case Control.

^b^N/A: not applicable.

Participants who gave permission to be recontacted during the original DeCC study were considered eligible for this study. Electoral rolls and social media were searched to obtain participants’ current contact details, and death records were checked to remove those deceased.

Invitation letters were sent to 511 depression cases and 587 controls whose addresses were known. The letters, with log-ons and the website address, were sent out in waves of approximately 200 with a follow-up letter or email a week later during February-December 2016. There were 142 returned as not known at that address, and 127 controls and 75 recurrent depression cases were successfully recruited. Assistance with Web-based completion of CLEAR was offered to 4 respondents who needed aid, from a researcher who visited the respondents with a Wi-Fi-enabled laptop. There was no notable difference in the responses from these participants.

The transition to university is associated with a large amount of life change [30], and students have high rates of depression [31]. Therefore, Middlesex University students were also recruited, mainly from first-year undergraduate psychology. The students were sent an email containing a log-on and the website address; 31.0% (126/406) responded from February- October 2016. A further 7 participants were recruited from the psychology department by convenience sampling.

There were 54 participants who started CLEAR but did not complete it. However, this was not considered problematic, as the timing suggests it was owing to difficulties with site loading, which occurred soon after 1 wave of letters was dispatched. This is supported by the fact that noncompleters were equally distributed between the case and control groups. None of the students failed to complete CLEAR, and recruitment procedures were halted during this time.

Ethical approval was granted from Middlesex Psychology Department’s Ethics Committee and Integrated Research Application System National Health Service ethics.

### Ethical Standards

The authors assert that all procedures contributing to this work comply with the ethical standards of the relevant national and institutional committees on human experimentation and with the Helsinki Declaration of 1975, as revised in 2008.

### Measures

#### Computerized Life Events Assessment Record Web-Based System

CLEAR mainly identifies life events and difficulties from the original LEDS interview [10], with updates to include a few new technological events (eg, cyber-fraud) and geopolitical circumstances (eg, asylum experience). It collects quantitative and qualitative data regarding demographics (eg, date of birth, partner status, and employment), information about close others (eg, relationship and frequency of contact), life events, and long-term problems. Additional questionnaires included the List of Threatening Experiences Questionnaire (LTE-Q) and General Health Questionnaire.

Life events and long-term problems are subsumed under 3 overarching groups, each of which is organized into several categories based on the LEDS domains: Lifestyle (education, work, housing, money, crime, and geopolitical events), Health (illness, pregnancy and bereavement), and Relationships (partner, children, and close others). Within each of these 12 categories, a list of potential events are presented, and if selected, the participant can use menus to further refine and add associated contextual information (Spence et al) [16]. The event type and context options are based on the precedent examples outlined in the LEDS manual. Participants are provided with guidance on rating 12-month life events and long-term problems consistent with the LEDS through written and video instruction, examples, and benchmarking. Throughout CLEAR, text fields for open responses and pull-down menus and checkboxes for closed responses are used. Life event short-term and long-term severity are rated from 1: “Extremely: life-changing, catastrophic, traumatic” to 5: “Not at all: no negative implications experienced or expected.” Focus describes who or what the event is focused on and is categorized as 1: “Self”, 2: “Joint”, 3: “Other,” or 4: “Possession.” Severe events are those rated ≥3 and are focused on the respondent either jointly (respondent and someone close) or alone (ie, rated 1 or 2). For all events, characteristics such as loss and danger are rated. A personalized feedback report is provided on life events and symptoms on completion.

#### The List of Threatening Experiences Questionnaire

The LTE-Q [32] is a self-report questionnaire comprising a list of 21 potentially significant life events to self or those they consider close (eg, family members) . This has been validated against the LEDS and used extensively, including with the DeCC sample previously [33]. It yields a score of the number of severe life events in the past 12 months.

#### The Life Events and Difficulties Interview

The LEDS [10] is an investigator-led semistructured interview of life events and difficulties. It includes extensive demographic information and covers 10 life event domains: education, work, fertility, crime, housing, money, health, other relationships, partner, and miscellaneous (including death, geopolitical events, etc). Information is collected on the event timing, surrounding context, the focus of the event (ie, who the event mainly affected), and other factors. Life event severity is rated on a 5-point scale (1 “marked”’ to 5 “not at all”) with higher points (1-3) required for a severe life event definition. The original scale was 4-point but supplemented by an additional scale of “a=upper” or “b=lower” for those rated “moderate” severity. These were subsumed into the adapted scale. Severe life events also require the *focus* of the event to include the “self,” either solely or “jointly” with another close person.

Difficulties (renamed Long-term Problems on CLEAR) were chronic stressors identified in main categories (eg, health, education) and rated on a 1-4 scale with severe difficulty (1=high marked, 2=low marked, and 3=upper moderate) and nonsevere (4=lower moderate). The interviews were conducted by RS or LK, and all LEDS interview ratings were checked by 1 of the original authors of the LEDS manuals (AB) blind to the study group and depression status, with queries reconciled at a consensus meeting of the 3 trained raters.

#### The General Health Questionnaire

The 12-item General Health Questionnaire [34] is a self-report symptoms questionnaire for depression that includes 6 positively worded and 6 negatively worded items rated along a 4-point Likert scale. Each question is dichotomized, with items denoting a greater frequency of symptoms (eg, “more so than usual” scored 1, and lower frequency ratings, eg, ”much less than usual” scored “0”). A score of 5 or more was taken to indicate a likely clinical case of depression [35]. The date of onset and peak symptoms was ascertained.

#### Data Analysis

CLEAR data were downloaded from MySQL and transformed into derived variables using Python programming language. The data were transferred into SPSS for statistical analyses. Group differences were assessed using chi-square analysis. Mann-Whitney U tests were used when the data were skewed.

Guidelines for reporting reliability and agreement studies [36] were followed. Cohen’s kappa (Κ) for dichotomous variables or intraclass correlation coefficients (ICCs) were used to assess the association over repeat testing and interview-CLEAR association, with the interpretation of the level of association guided by Cohen’s accepted levels [37]. Analyses focused on severe life events and long-term problems, as these are most pertinent to clinical and research use.

The sensitivity (true positive) and specificity (true negative) of CLEAR in comparison with LEDS was calculated for severe life events. Sensitivity reflects the ability of a test to correctly classify when the property of interest is present (true positive), whereas specificity indicates the ability of a test to correctly classify when it is absent (true negative). Logistic regression was used to examine severe life events in relation to depression.

## Results

### Prevalence of Life Events

In the sample as a whole, the average rate of CLEAR life events was 2.28 (SD 2.37, range 0-8), with 41.5% (136/328) of the sample having at least 1 severe life event (Table 2). For long-term problems, the average was 1.28 (SD 1.99, range 0-19), with 49.7% (163/328) of the sample having at least 1 long-term problem, and 32.0% (105/328) having at least 1 severe long-term problem. Table 2 shows comparisons between the subgroups. The clinical group had significantly more severe life events, long-term problems, and severe long-term problems. The students had significantly fewer life events, long-term problems, and severe long-term problems than the DeCC group. The 2 DeCC groups did not have significantly different LTE-Q scores, but students reported significantly more LTE-Q events.

### Test-Retest Reliability

In total, 173 life events were reported; 53 events were rated as severe at either 1 or both time-points, and 15% (9/61) individuals reported no events at either time-point. There was good test-retest agreement for severe life events (85.4%, Κ=.60, 95% CI 0.40 to 0.81; *P*<.001). The reliability of severe life event characteristics reported using CLEAR is shown in Table 3.

The association between the overall number of events at both time-points was good for CLEAR (ICC=.89, 95% CI 0.82 to 0.94); however, this did vary by domain, ranging from.93 (partner) to.28 (money). The association was moderate at retest for the LTE-Q (ICC=.75, 95% CI 0.56 to 0.86) with ICCs ranging from.85 (separated from partner) to.17 (Burglary or mugged).

There were 94 long-term problems reported, and 22/61 (36%) participants reported no long-term problem at either time-point. The agreement for severe long-term problems was modest (Κ=.38, 95% CI 0.21 to 0.55; *P*<.001). Table 3 shows associations for characteristics of severe long-term problems at both time-points.

**Table 2:** Life event and long-term problem frequency by group (N=328).

Life events and long-term problem	Total DeCC^a^ (n=202), mean (SD); range	Controls (n=127), mean (SD); range	Clinical (n=75), mean (SD); range	Mann-Whitney U Test	*P* value	Students (n=126), mean (SD); range	Mann-Whitney U Test	*P* value
Life events	2.41 (2.28); 0-11	2.11 (1.88); 0-10	2.91 (2.77); 0-11	1.70	.09	2.08 (2.50); 0-12	1.97	.049
Severe life events	0.78 (1.28); 0-8	0.50 (0.82); 0-3	1.25 (1.73); 0-8	3.38	.001	0.75 (1.14); 0-6	−0.01	.99
Long-term problems	1.57 (2.24); 0-19	1.01 (1.43); 0-7	2.53 (2.94); 0-19	4.82	.001	0.80 (1.40); 0-7	3.89	.001
Severe long-term problems	0.75 (1.31); 0-7	0.37 (0.70); 0-3	1.39 (1.79); 0-7	4.82	.001	0.42 (0.96); 0-5	2.76	.01
List of Threatening Experiences Questionnaire events	3.83 (3.18); 0-15	3.38 (2.67); 0-13	4.59 (3.80); 0-15	1.88	.06	5.07 (3.01); 0-16	−4.17	.001

^a^DeCC: Depression Case Control.

**Table 3:** Test-retest reliability of severe life events and long-term problems’ attributes.

Attributes		Intraclass correlation coefficient	*P* value
**Severe life event attribute**			
	Category	.91	.001
	Focus (1-4)^a^	.64	.01
	Short-term threat (1-5)	.42	.09
	Long-term threat (1-5)	.63	.01
**Long-term problem attribute**			
	Category	.97	.001
	Who involved	.65	.002
	Severity now (1-4)	.70	.001
	Severity worst (1-4)	.62	.01

^a^Focus describes who or what the event is focused on and is categorized as 1: “Self”, 2: “Joint”, 3: “Other,” or 4: “Possession.”

### Concurrent Validity of Computerized Life Events and Assessment Record and Life Events and Difficulties Schedule

Across CLEAR and LEDS, 184 life events were reported, of which 72 were rated severe on 1 or both measures. Owing to missing data, analyses could only be conducted for the events recorded by both measures (48/184, 26.1% of all events). The level of agreement for severe life events was fair but not significant (Κ=.25, 95% CI −0.02 to 0.52, *P*=.09). Both specificity and sensitivity for severe events were moderate (65.4%, 95% CI 44.3 to 82.8 and 59.1%, 95% CI 36.4 to 79.3, respectively). The characteristics of events were examined across LEDS and CLEAR (Table 4).

There were 88 long-term problems recorded, 47 severe ratings were given, and 4/30 (13%) respondents reported no long-term problems on either measure. As with the events, only the minority of long-term problems were captured by both methods (21/88, 24%), and therefore, owing to missing data, analyses could only be performed on these. The agreement for severe long-term problems was moderate (Κ=.43, 95% CI 0.05 to 0.81, *P*=.04), but the sensitivity (66.7%, 95% CI 34.9 to 90.1) and specificity (77.8%, 95% CI 40.0 to 97.2) were good.

### Concurrent Validity of List of Threatening Experiences Questionnaire and Computerized Life Events and Assessment Record

Severe life events on CLEAR and LTE-Q were compared for the total sample of 328. There was poor agreement (Κ=.06, 95% CI 0.01 to 0.11, *P*=.03) owing to many more events being identified only by the LTE-Q (n=170, 52%). Sensitivity was 43.1% (95% CI 37.5 to 48.9) and specificity was 78.6% (95% CI 59.1 to 91.7).

### Relationship Between Computerized Life Events and Assessment Record, Severe Life Events, and Depression

The presence of at least 1 severe life event in CLEAR related to depression: 41.4% (55/133) of those with a severe life event were depressed versus 16.8% (32/191) of those with no severe life events (odds ratio, OR 3.50, 95% CI 2.10 to 5.85; *P*<.001). This held in the DeCC clinical group (OR 3.45, 95% CI 1.30 to 9.15, *P*=.01) and the student group (OR 3.62, 95% CI 1.68 to 7.80; *P*<.001) but not the DeCC control group (OR 2.11, 95% CI 0.58 to 7.73, *P*=.26), where both severe life events and depression were at a low rate. The majority of domains with 10 or more severe life events also significantly predicted depression (Table 5).

**Table 4:** Concurrent validity; Life Events and Difficulties Schedule Interview versus Computerized Life Events and Assessment Record characteristics of events (N=48).

Variable	Intraclass correlation coefficient	*P* value
Category	.85	.001
Focus	.91	.001
Short-term severity (1-5)^a^	.52	.01
Long-term severity (1-5)^a^	.49	.01

^a^Life event short-term and long-term severity are rated from 1: “Extremely: life-changing, catastrophic, traumatic” to 5: “Not at all: no negative implications experienced or expected.”

**Table 5:** Computerized Life Events and Assessment Record Severe Life Events by category and General Health Questionnaire depression.

Computerized Life Events and Assessment Record event category	Severe life event, n/N (%) depressed	No severe life event, n/N (%) depressed	Odds ratio (95% CI)	*P* value
Education	19/33 (57.5)	68/291 (23.4)	4.45 (2.12-9.35)	.001
Work	17/36 (47.2)	70/288 (24.3)	2.79 (1.37-5.65)	.01
Housing	6/10 (60.0)	81/314 (25.8)	4.32 (1.19-15.68)	.03
Money	8/17 (47.1)	79/307 (25.7)	2.57 (0.96-6.88)	.06
Health or death	27/66 (40.9)	60/258 (23.3)	2.29 (1.29-4.04)	.004
Partner	7/16 (43.7)	80/308 (26.0)	2.22 (0.80-6.15)	.13
Other relative	9/16 (56.2)	78/308 (25.3)	3.79 (1.37-10.52)	.01

The presence of a provoking agent was examined in relation to the onset of depression. This required the selection of the severe life event immediately prior to the onset of disorder or severe life event closest to the point of CLEAR completion for those not depressed. This excluded severe events during or after the depression. This showed that 36.1% (44/122) of those with a provoking agent had depression vs 21.3% (43/202) with no provoking agent (OR 2.09, 95% CI 1.27 to 3.44, *P*=.004).

The presence of a severe long-term problem was similarly related to depression, with 44.1% (45/102) with a severe long-term problem reporting depression versus 18.9% (42/222) without a severe long-term problem (OR 3.38, 95% CI 2.02 to 5.67; *P*<.001). This relationship held in the DeCC clinical group (OR 4.0, 95% CI 1.48 to 10.80, *P*=.01) and the student group (OR 3.67, 95% CI 1.55 to 8.70, *P=*.003) but was nonsignificant in the DeCC control group (OR 1.98, 95% CI 0.52 to 7.5, *P*=.32).

When LTE-Q events were grouped by category, none were statistically related to depression (Health OR 1.39, *P*=.29; Work OR 1.35, *P*=.29; Crime OR 1.26, *P*=.40; Fertility OR 1.62, *P*=.09; Housing OR 1.42, *P*=.17). The presence of any 1 severe life event similarly did not relate to depression: 27.0% (80/296) of participants with an LTE-Q event versus 25.9% (7/27) without an LTE-Q event were depressed (OR 1.06, 95% CI 0.43 to 2.60, *P*=.90). However, there was a modest association between LTE-Q score and General Health Questionnaire symptom score (r=0.19; *P*<.001).

## Discussion

### Summary of Results

The results demonstrated that CLEAR significantly predicted depression and was superior to a commonly used checklist approach. The test-retest reliability was good for severe life events and their characteristics, although agreement missed the significance for short-term threat ratings. Reliability was fair for severe long-term problems and good for their characteristics. In comparisons with the LTE-Q, CLEAR performed better.

Although the average rate of life events found by CLEAR was similar to previously reported interview [10] and self-report [12] rates, CLEAR missed the majority of life events and long-term problems rated by LEDS. However, it is likely this is because the LEDS records many more nonsevere events; in the validity sample, 26.9% of LEDS events were severe, compared with 46.2% of CLEAR events. Additionally, the LEDS events could often be trivial in nature (eg, “*husband started TEFL course,” “* *end of module* *exams”*). Furthermore, each LEDS event was rated separately, for example, “*job interview”* and “*starts new job”* would be recorded as 2 events, whereas in CLEAR, these were likely collapsed into 1. Perhaps discrepancies could be reduced by having more active rather than passive prompts for events throughout CLEAR.

Nevertheless, for the events that were captured, the results were promising. The specificity and sensitivity for severe events were moderate, and the event characteristics had fair to very good associations. Severe long-term problems also had a moderate agreement, sensitivity, and specificity. Crucially, predictive validity showed a high association between CLEAR severe life events and depression, including those prior to onset, consistent with prior research [4,38] and superior to the checklist findings.

### Implications

The issue of a lower event and long-term problem identification in CLEAR when compared with LEDS needs to be considered in relation to its potential usefulness. Where event totals are the key element, the method would miss many potential events, although still have more potential coverage than checklist approaches. However, for clinical purposes, CLEAR’s more robust inclusion of severe events and the significant associations with depression indicate greater utility than self-report checklists. The tool could aid with the routine assessment of stressors where these relate to disorder or treatment outcomes. For instance, identifying key provoking agents in emotional or trauma-related disorders to be linked with cognitive behavioral therapy treatment or identifying the number and range of severe stressors relevant for lifestyle risks in health settings such as antenatal care or diabetes treatment. Indeed, CLEAR can be personalized with different outcome measures and respondent feedback, making it a flexible measurement tool.

### Limitations

Nonetheless, there are limitations to this study. The sample was skewed by age and gender and is not representative. The actual response rate was not calculated owing to the lack of information on the accuracy of the DeCC sample contact details, and a proportion did not complete CLEAR. The self-report symptom scale is only a proxy measure of clinical depression. Finally, the validity subsample was rather small and proved insufficient for comprehensive validation of long-term problems.

### Strengths

Despite this, CLEAR is a promising tool for assessing life stress in large, nationally distributed samples including gene- environment research, which requires large numbers. Here self-report measures have been found to be less effective [13] and face-to-face interviews impractical. CLEAR is quick and cheap to administer, and the reliability and validity were shown to be good for depression-related events (those severe and focused on the individual). Moreover, the automated coding to provide prederived SPSS variables enables future ease of data analysis. The measure is likely to be effective for the large-scale study of depression and other disorders involving severe life events.

### Conclusions

The study indicates success in producing a more sophisticated measure of socioenvironmental stressors with the use of new technologies. CLEAR is a viable option for clinical or research services wanting to provide more exact predictions of risk to help prevent and treat disorders.

## Abbreviations

CLEAR: Computerized Life Events and Assessment Record
DeCC: Depression Case Control
ICC: intraclass correlation coefficient
LEDS: Life Events and Difficulties Schedule
LTE-Q: List of Threatening Experiences Questionnaire
OR: odds ratio
